# 

**Published:** 1896-09

**Authors:** 


					CONTENTS.
The Causation and Treatment of
Sudden Dyspnoea in Goitre.
By
Charles A. Morton, r.K.C.b.
Eng   ...
213
A Case of Hystero-Epilepsy
By
Bonville B. Fox, M.A., M.D. Oxon.
On the Use of Oxygen in the Treat-
ment of Anaemia, with a Case
Treated by Transfusion of Blood.
By J. Michell Clarke, M.A., M.D.
Cantab., F.R.C.P. Lond
232
A Case of Needle in Foot Revealed by
the X Rays.
By W. E. Pountney,
M.D
238
Progress of the Medical Sciences:
Medicine.
Bv R. Shingleton Smith,
M.D.
240
Surgery.
By W. H. Harsant
245
Dermatology.
By Henry Waldo,
M.D.
Jenner
255
Reviews of Books
256
Notes on Preparations for the Sick
277
The Library 01 the Bristol Medico-
Chirurgical Society 281
Local Medical Notes
284
Scraps

				

